# The F1000Research Antibody Validation Article Collection

**DOI:** 10.12688/f1000research.5405.2

**Published:** 2014-11-11

**Authors:** Matthew A. Helsby, Mei Yee Leung, Andrew D. Chalmers

**Affiliations:** 1 CiteAb Limited, Bath, BA1 1BE, UK; 2 St John’s Laboratory, London, SE8 5RT, UK; 3 Department of Biology and Biochemistry, University of Bath, Bath, BA2 7AY, UK

## Abstract

Well validated antibodies are crucial to progress in a wide range of life science disciplines, but validating an antibody is a complex and ongoing process. Antibody validation is often carried out as preliminary work to a larger study so the validation data may go unpublished and needless duplication of efforts can occur. This collection of articles in *F1000Research* provides a home for papers describing antibody validation studies. Our goal is to encourage publishing of all studies, both positive and negative, which increase understanding of how antibodies perform. These could range from large studies with thousands of antibodies to small single figure studies which validate an individual antibody for a specific purpose. Opinion or Correspondence articles considering any aspect of antibody validation are also welcome. Here, we provide an introduction to the collection which we hope will grow and become a valuable resource for the many thousands of researchers who use antibodies.

## Editorial

Well validated antibodies are crucial to enable scientists to make progress in a wide range of life science disciplines ranging from Neuroscience to Tissue Engineering to Plant Science. Evidence of validation is important as it allows scientists to choose antibodies that are fit for their experiments and avoid wasting time optimising antibodies that are unsuitable. Validation data also provides reviewers a guide as to whether the antibodies used in a manuscript are likely to give reliable results, something which helps to ensure experimental reproducibility, a topical issue in today’s life sciences.

Validating an antibody is a complicated process that can involve many different approaches ( Bordeaux *et al.*, 2010; Howat *et al.*, 2014). Historically antibody validation commonly involved the now controversial antigen pre-adsorption test ( Holmseth *et al.*, 2012), while current studies may make use of knockout or knockdown tissue to demonstrate specificity. There are also large scale approaches, capable of validating many antibodies simultaneously ( Holm *et al.*, 2012). However, there is no simple experiment that can validate an antibody for all possible applications and samples. For example, validating an antibody for western blotting using a human cell line, does not guarantee the antibody will be suitable in immunohistochemistry using tissue from a rat. Instead antibody validation is a gradual process which involves testing the antibody for specific applications and species/tissues of interest, ideally using a number of approaches.

This collection provides a home for papers describing antibody validation studies. Our aim is to encourage publishing of all studies, both positive and negative, which increase understanding of how antibodies perform. These can range from large studies involving hundreds of antibodies, or the use of many tissues or cell lines, to small single figure studies focusing on an individual antibody in a specific setting. The studies should have been sufficiently repeated and the results accurately and fully reported. It is also crucial that the Materials and methods provide enough detail to allow the experiments to be reproduced, something which is often not the case with studies using antibodies ( Helsby *et al.*, 2013). A key part of ensuring reproducibility is to make sure the antibodies can be identified by including their supplying company name and code and a resource identifier issued by the Research Identification Initiative ( http://scicrunch.com/resources).

The instructions to authors ( Box 1) and guidelines for reviewers have been tailored to facilitate the aim of encouraging a broad range of papers, with a focus on reproducibility and accurate reporting, rather than perceived impact. Correspondence and Opinion articles on any aspect of antibody validation are also welcomed.

**Box 1. Extract from the Instructions to Authors**

*The Antibody validation article collection aims to provide a platform for antibody validation studies and enhance the reliability and reproducibility of antibodies in scientific research. Referees reviewing validation studies will not focus on novelty and impact, but rather on whether the study is scientifically sound and provides all relevant information.*

*F1000Research accepts a variety of validation studies, which will be published as Research Notes:*

- *New antibodies; either against a new target or a new antibody raised against an existing target.*
- *New applications for existing antibodies; either in a new biological system or a new application tested within an existing/previously tested biological system.*
- *Existing antibody applied to a new biological system; new organism/tissue/cell type.*
- *Validations of previously tested antibodies that are carried out in more depth than before, in one or more applications.*
- *Validations of groups of antibodies raised against the same target.*
- *Antibodies that failed to meet the validation criteria.*
- *Replication studies that confirm or disagree with previously published validations.*

This broad approach should encourage a wide range of studies, many of which may never be published without this initiative and we hope that as the collection grows it will become a valuable resource for the thousands of researchers who use antibodies.
